# Development and Validation of a Motivation Scale for Status Pursuit by Organization Members

**DOI:** 10.3389/fpsyg.2022.844325

**Published:** 2022-07-14

**Authors:** Zhicheng Wang

**Affiliations:** ^1^School of Economics and Management, Hainan Normal University, Haikou, China; ^2^School of Business, Jiangxi University of Finance and Economics, Nanchang, China

**Keywords:** scale development, organizational member status, regulatory focus theory, promotion-focused self-regulation, prevention-focused self-regulation

## Abstract

Based on a literature review and questionnaire survey, the study proposes two tendencies of employees’ status pursuit motivation. It applies exploratory factor analysis, confirmatory factor analysis, and other statistical methods to develop a scale of motivation for status which contains nine items and two dimensions. The development stage of this scale consists of the generation of the initial scale, exploratory research and verification research. In the generation stage of the initial scale, we obtained 12 initial questions of the status scale by combing status literature and adopting open questionnaire among employees of knowledge-based enterprises, and determined 10 of them as the questions of the initial scale by text analysis method. In the exploratory research stage, we conducted pre-survey and formal questionnaire collection on some enterprises in Guangdong and Jiangsu provinces of China. In the pre-survey stage, we distributed 100 questionnaires (81 were recovered), and the pre-survey results were satisfactory. Accordingly, 400 questionnaires were distributed (370 were recovered). In the exploratory research stage, we verified the organization member status pursuit motivation scale consisting of two dimensions and nine items. In the stage of validation research, 450 questionnaires were distributed to knowledge-intensive enterprises in Guangdong province, Jiangsu Province, Jiangxi Province and so on (425 were returned). The validation research on organizational membership motivation scale included violation estimation test, confirmatory factor analysis, model fit evaluation, reliability and validity test. Finally, a scale of status pursuit motivation of organization members consisting of two dimensions and nine items is obtained. This study expands the measurement methods of status research from a new perspective and lays a foundation for subsequent empirical research on organizational member status pursuit motivation.

## Research Background

Status is a basic motivation of human activities and has been an important research topic in the fields of sociology and psychology for some time. However, status has not achieved the same “status” in the field of management, as it has not been valued by management scholars (Peterson, 2012) until recent years. Status is a kind of social evaluation concept. Organizational membership status refers to the relative ranking of employees in the organization and is the subjective evaluation of their prestige, respect, and excellence by other members of the organization (Huberman et al., 2004; Anderson and Kilduff, 2010; Djurdjevic et al., 2017). Obtaining a higher status can bring many benefits. For example, the promotion of status will make the members of the organization obtain more opportunities for success, more happiness, less stress, and better physical and mental health (Anderson et al., 2015). Therefore, within an organization, members have the motivation to gain higher status and gain more benefits through the promotion of their status (Bendersky and Shah, 2013; DesJardins et al., 2015).

According to the status characteristics theory, social status refers to an individual’s position in a social system based on their characteristics, assets, and behaviors. Thus, social status itself is an incentive mechanism that can affect judgment and decision making (Weiss and Fershtman, 1997). The acquisition of status can be divided into ascribed status and achieved status. Ascribed status originates from demographic characteristics such as gender, race, origin, etc. This status usually involves social prejudice and stereotyping, low controllability, high stability, and other characteristics, and there is also ascribed status within the organization. One study found that ascribed status has both a direct and an indirect impact on individual final acquisition status (Blalock et al., 1967). In addition, Lin (2001) stated that human capital (education and experience), initial status (parental or previous professional status), and the social connections of the individual self (such as the breadth of connections) determine the social resources that the individual can access through their relationships, while the social resources available to the individual affect their acquisition status such as professional status, authority status, department, or income. Achieved status is mainly related to certain factors such as education, occupation, and marital status, as well as certain of the individual’s active choices. The cultural values of achieved status response are mainly derived with high variability from achievements that individuals can control, such as pursuing higher education, providing interpersonal help, improving job performance, etc. (Phillips and Dumas, 2009; Hogue et al., 2011; Howell et al., 2015; Wei et al., 2017). According to the status characteristics theory, scholars have defined the ways individual status can be obtained and classified the two ways of acquiring individual status. However, under this theoretical framework, prior studies have not explained the intrinsic motivation of, and differences in, individuals’ pursuit of status.

The pursuit of status is one of the most important basic motivations of human beings. People engage in every act in order to obtain or maintain status (Chen et al., 2012). Status concern is widespread in different cultural contexts, but there are individual differences in people’s attitudes and motivations toward the pursuit of status. The pursuit of different statuses and goals will affect employees’ behavior choices. Hu and Xie (2015) state that in the field of knowledge management, knowledge hiding is mainly motivated by status preservation, while knowledge sharing is mainly motivated by status improvement (Park J. et al., 2017). However, due to the lack of a dimensional structure or measurement tools for status pursuit motivation, this view has not been empirically demonstrated. According to the different content of status requirements, Liu et al. (2013) divides status competition motivation into dominance-based status-striving motivation and prestige-based status-striving motivation. The former refers to the expectation to control resources and others, which is a typical pursuit of possession. The latter expects others to recognize, accept, and strive to maintain their “perfect” image in the eyes of others, which is a typical symbolic pursuit. Both of these have a significant positive effect on innovative behavior. Other studies have found that to pursue status, employees may commit immoral behaviors such as deception (Pettit et al., 2016), and for the motivation of improving status, employees may put resources for personal performance into status-seeking activities, resulting in a decline in individual performance (Bendersky and Shah, 2012). The inconsistency of existing research conclusions requires us to further explore the internal structure of status pursuit motivation to clarify the real relationship between status pursuit motivation, job performance, and other outcome variables.

## Theoretical Basis

For a long time, empirical research on organizational membership status has mainly been carried out within the background of Western culture. In recent years, membership status in the organization has become a topic of wide interest by scholars at home and abroad. In non-Chinese academic circles, early studies on organizational hierarchy mixed status with power, but with the deepening of research, more and more empirical studies have shown that status and power are two completely independent psychological constructs in terms of formation basis and mechanism of action. Some scholars have explored in detail the acquisition, maintenance, and experience of organizational membership status in the workplace (Loch et al., 2000; Blader and Chen, 2012; Chen et al., 2012; Anicich et al., 2015; Hays and Bendersky, 2015; Smith and Magee, 2015). Membership status in organizations has gradually moved to the forefront of organizational behavior research. In China, research on membership status in organizations has gradually increased in importance. At present, scholars have examined the relationship between status and knowledge sharing and innovation, and have made a detailed review of the research on membership status in Western organizations (Liu et al., 2014, 2015; Wei and Zhang, 2014; Hu and Xie, 2015; Wang and Du, 2015; Wei et al., 2015). Hu and Xie (2015) showed that in the field of knowledge management, knowledge hiding is mainly motivated by the status preservation of organization members. Liu et al. (2013) based on different organization members’ demands for status, divided status competition motivation into dominance-based status-striving motivation and prestige-based status-striving motivation. Dominance-based status-striving motivation refers to the expectation of controlling resources and others, which is a typical possession pursuit. Prestige-based status-striving motivation is a typical symbolic pursuit, expecting others’ recognition and acceptance and striving to maintain a “perfect” image in the eyes of others. Research shows that both of these have significant positive effects on innovation behavior. Wei et al. (2015) systematically reviewed organizational hierarchy from the perspective of Confucian hierarchy. Wang and Du (2015) summarized the connotation, characteristics, and measurement methods of organizational status, distinguished organizational status from organizational reputation, and summarized the evolution mechanism of organizational status. Existing literature, however, has not distinguished between maintenance status and status of motivation: they are collectively referred to as status or care status, and the empirical study found in the maintenance of status and obtain the position, driven by people will take different behavioral responses, with the deepening of the research group status, the research conclusions are inconsistent. In addition to the above positive effects of status pursuit, other studies have found that status pursuit may lead employees to engage in unethical behaviors such as cheating (Pettit et al., 2016). The motivation of improving status may lead employees to invest resources used for personal performance into status pursuit. As a result, individual performance declines (Bendersky and Shah, 2012). The above studies show that status pursuit is a basic motivation of people’s activities, but status pursuit motivation is not a one-dimensional construct. Future research needs to further explore the internal structure of status pursuit motivation, so as to clarify the real relationship between status pursuit motivation and job performance and other outcome variables.

As a new approach to human motivation, regulatory focus theory examines how individuals avoid undesired end-states and approach the desired end-states. The so-called “regulatory focus” refers to the specific ways individuals use self-regulation to achieve goals (Higgins, 1997). According to regulatory focus theory, there are two basic self-regulation systems: prevention-focused self-regulation and promotion-focused self-regulation. Both of these are necessary for human survival and neither is good or bad (Higgins, 1997). Prevention-focused individuals focus on obligations and responsibilities. They avoid negative outcomes and tend to be conservative. Promotion-focused individuals are mainly concerned with achievement and desire fulfillment. They are more proactive, desire to achieve positive goals, and like to engage in adventurous activities. According to the regulation focus theory (Higgins, 1997, 1998), any goal can be achieved through different strategic means (Gamache et al., 2015). Individual focus orientation, for example, different attitude toward knowledge transfer activity has a distinct. Regulatory focus theory provides a good explanation for the role of organization member status as motivation. In the relationship between organizational membership status and knowledge sharing, promotion-focused employees are more inclined to adopt behaviors beneficial to the organization such as knowledge sharing in pursuit of a dominant position in the organization or in expectation of obtaining more resources (such as knowledge and information) (Kark and Dijk, 2007; Liu et al., 2013). By contrast, prevention-focused employees tend to hide knowledge in order to avoid losing their current advantages and competitiveness in the organization (Connelly et al., 2012). Therefore, we believe that the regulatory focus theory can well explain the different choices of organizational members to pursue status under different motivations.

Regulatory focus theory differentiates two kinds of regulatory focus, deepens our understanding of individual behavioral motivation, and is regarded as an emerging motivation theory (Mao, 2017). According to regulatory focus theory, there are two kinds of regulatory focus in the process of achieving goals: prevention-focused self-regulation and promotion-focused self-regulation. The promoters tend to adopt aggressive ways to achieve goals. They are willing to take risks and even carry out immoral behaviors more often. Prevention-focused people tend to achieve their goals in an evasive manner. They like stability and are comfortable with the *status quo* (Gino and Margolis, 2011; Wei and Zhang, 2014). Anderson et al. (2015) stated that there are individual differences in the purpose of status pursuit. Hu and Xie (2015) presented a reasonable solution to the dual motivation problem: status difference brings both status improvement (promotion) and status preservation (maintenance) to team members. They did so by introducing a moderating variable (status stability). However, their contribution still fails to solve the fundamental problem of measuring organizational member status motivation in different dimensions. In light of the regulatory focus theory, we infer that status-seeking motivation has a potentially multidimensional structure. Specifically, status-seeking motivation may include two dimensions: prevention-focused status pursuit motivation and promotion-focused status pursuit motivation, but its actual structural dimension needs to be obtained through a standardized scale development process.

## Method and Results

### Concept Definition

Studies on the formation mechanism of member status within an organization are mainly carried out within the framework of status characteristics theory (Berger et al., 1972; Webster and Driskell, 1978; Bianchi et al., 2012). According to the status characteristics theory, status can be divided into ascribed status and achieved status. Prior studies have seldom considered individual differences in status pursuit. From the perspective of the influence results of the researches on the status of members within an organization, non-Chinese scholars have concluded that status based on competency is linked to individual competency, and is thus more likely to bring positive results. Dignity-based status, by contrast, is often unrelated to personal competency (Fast et al., 2012; Wei et al., 2017). Its consequences are often neutral or even negative (Fast et al., 2012; Bendersky and Shah, 2013). Some scholars have concluded that prestige-based status-striving motivation has a positive impact on organizational behaviors such as innovation (Liu et al., 2013). Other studies have concluded that members of organizations may engage in unethical behaviors such as cheating in the pursuit of status (Pettit et al., 2016), or invest personal resources in status pursuit activities. This leads to the negative consequences of individual performance decline (Bendersky and Shah, 2012). The inconsistencies in the existing research on the impact of organizational membership status on outcomes suggest that there are individual differences in the motivations for organizational membership status pursuit, which will lead to different outcomes. These inconsistencies in the existing research conclusions require us to further explore the internal structure of status pursuit motivation (Wang et al., 2020).

According to the regulatory focus theory, individuals have two basic self-regulation systems: prevention-focused self-regulation and promotion-focused self-regulation. Based on regulatory focus theory, this paper argues that there may be two motivations, prevention and promotion, in the pursuit of organizational status; these motivations are neither good nor bad. This is potentially a useful way to solve the problem of individual differences in status motivation. That is to say, the motivation of status pursuit by organization members refers to the specific tendencies shown by individuals in the process of self-regulation in the pursuit of organizational status. These two tendencies are manifested as prevention-focused status pursuit motivation and promotion-focused status pursuit motivation, respectively.

Specifically, members of an organization who are motivated by promotion-focused status pursuit have the need to grow, improve, and develop (Tumasjan and Braun, 2012). Individuals motivated by promotion status pursuit orient themselves toward their ideal self, and their behavioral strategy is to narrow the gap between their current state and their ideal state through self-regulation (Park T. Y. et al., 2017). In order to pursue a more solid organizational position, individuals in this state pay attention to and desire achievement. They are more active, eager to achieve positive goals, and like to engage in adventurous activities. Organization members with a prevention-focused status pursuit motivation have a need for security and stability in the pursuit of motivation (Tumasjan and Braun, 2012). Individuals with a prevention-focused status pursuit motivation orient themselves toward the ought self (such as individual responsibilities and obligations), and their behavioral strategy is to narrow the gap between the real state and the ought state through self-regulation (Park T. Y. et al., 2017). In order to maintain their own status, members of organizations with prevention-focused status pursuit motivation pay more attention to obligations and responsibilities. They avoid negative results and tend to act in a conservative fashion. Based on regulatory focus theory, we developed a scale from the dimensions of prevention-focused status pursuit motivation and promotion-focused status pursuit motivation.

### Creation of the Initial Scale

Considering the continuity of the scale development process and the uniqueness of this study’s theoretical perspective, we began by extensively reading the core journals related to organizational status measurement and status research at home and abroad in recent years. We did so to assess the *status quo* of status research to provide a reference and a basis for the smooth progress of this study. A questionnaire survey was then distributed to high-level, middle, and grassroots personnel of knowledge-intensive enterprises. From the prevention-focused perspective, the main questions regarding status pursuit motivation were: “What do you think you need to pay attention to in order to maintain your position in the organization?” and “What are the specific aspects of performance? Please list five to ten.” From the promotion-focused perspective, the main questions regarding status-seeking motivation included: “What do you think you need to actively do to achieve a higher organizational status?” and “What are the specific aspects of performance? Please list five to ten.”

Based on the above, a text analysis method was adopted to encode the collected data with declarative sentences as the analysis unit. After repeated deliberation by a three-member coding team, 33 initial concepts were finally extracted. After the initial statements were combined with similar and simplified items, 12 statements were obtained. The 12 sentences were printed on cards, and three sets of cards were prepared. One professor of management and two associate professors of management were invited to independently complete the classification, induction, and naming of the sentences. The results showed that the consistency of classification and induction was higher among the three. Specifically, there were 10 statements (83.3%) whose classification and induction results were completely and immediately consistent. The remaining two statements (16.7%) were divided into different categories and reached consensus after repeated discussion among the three people. Finally, the above statements were divided into two dimensions: prevention-focused status pursuit motivation and promotion-focused status pursuit motivation. This classification method is consistent with the definition of organizational membership status based on the moderating focus theory used in this study.

### Exploratory Study

#### Sample and Collection

The research team distributed questionnaires to knowledge-intensive enterprises in Guangdong province for exploratory research and reliability analysis of the scale used in this study. We promise participants that the data obtained will only be used for academic research and will not be used for other purposes, and that the confidentiality of the questionnaire will be guaranteed. As a preliminary survey of the initial scale, 100 questionnaires were issued and 81 were recovered. The analysis of pre-survey data showed that the results of item analysis, exploratory factor analysis, and reliability analysis of the initial scale were satisfactory. Based on the above results, we further conducted a questionnaire survey of employees of knowledge-intensive enterprises in Guangdong province and Jiangsu Province. A total of 400 questionnaires were sent out and 370 were recovered. After removing questionnaires with more than three missing items or more than five consecutive identical options, 349 questionnaires were finally obtained, with an effective recovery rate of 94.3%. As can be seen from Table 1, male employees accounted for 48.7%. In terms of age, 11.5% were 25 and below, 39.3% were from 26 to 35 years old, 36.4% were aged 36–45, and those over 46 years old accounted for 9.5%. In terms of educational level, 23.2% had a senior high school education or below. Vocational high school and junior college accounted for 26.6%. Undergraduates accounted for 41%. 9.2% had a master’s degree or above. In terms of working years, 10.3% had worked for 1 year or less. Two to five years accounted for 37.8%. More than 5 years accounted for 51.9%.

**TABLE 1:** Composition of valid samples (*N* = 349).

Name	Category	Percent	Name	Category	Percentage
Gender	Male	48.7	Education	High school or below	23.2
	Female	51.3		Vocational school/College	26.6
Age	25 years old	11.5		Undergraduate	41
	and below				
	26–35 years old	39.3		Master degree or above	9.2
	35–45 years old	36.4	Work	One year or less	10.3
	Over 46 years old	9.5		Two to five years	37.8
				More than 5 years	51.9

#### Measures

SPSS20.0 software was used to perform KMO and Bartlett sphere tests on the data to confirm whether exploratory factor analysis could be performed. The KMO statistic is 0.805, indicating that common factors exist between variables, which is suitable for factor analysis. The Bartlett sphere test conforms to the standard of significance level of 0.05, indicating that there are common factors among correlation matrices representing the whole population, which also indicates suitability for factor analysis. Factor extraction was carried out according to the principle of feature roots being greater than 1 and the skew rotation method. Two items, “I will work carefully and conscientiously” and “I will be honest and adhere to principles,” were deleted as they lacked a good identification degree. In addition, one item, “I will take the initiative to undertake difficult work,” was deleted because its factor load was less than 0.4. After several rotations, a scale for organizational member status pursuit motivation was obtained, which consisted of two dimensions and nine items. As can be seen from Table 2, the results of exploratory factor analysis are basically consistent with the pre-set factor results. Two dimensions of prevention-focused status pursuit motivation and promotion-focused status pursuit motivation were extracted, and the total variance explanation percentage reached 62.78%. This shows that the concept of the membership status pursuit motivation scale has been preliminarily verified. The results of confirmatory factor analysis are shown in Table 3. It can be seen from Table 3 that the fitting effect of the two-dimensional model is good.

**TABLE 2:** Exploratory factor analysis results (*N* = 349).

Item	Promotion-focused	Prevention-focused
A1 I will take the initiative to share my knowledge and skills	0.704	
A3 I will overfulfill the task assigned by the leader	0.767	
A4 I will take the initiative to improve the skills required by the work	0.863	
A5 I will take the initiative to help others	0.759	
A6 I will actively participate in the activities of the company	0.702	
A9 I will be reserved in my work		0.802
A10 I will not give opinions		0.839
A11 I will take my job seriously		0.846
A12 I will make sure I have the necessary skills for my job		0.804

**TABLE 3:** Results of confirmatory factor analysis (*N* = 349).

Model	χ2	df	RMSEA	GFI	CFI	RMR
One-factor model	622.833	27	0.252	0.670	0.485	0.261
Two-factor model	80.029	26	0.077	0.954	0.953	0.053

#### Data Analysis and Result

SPSS20.0 software was used to analyze the reliability of the sample data. The results showed that the α values of each dimension of prevention-focused status pursuit motivation and promotion-focused status pursuit motivation were 0.814 and 0.842, respectively, and the α values of each dimension of the prevention-focused status pursuit motivation and the promotion-focused status pursuit motivation were decreased after deleting any item. The α value of the status pursuit motivation scale for the whole organization was 0.746, which was higher than the standard of 0.7. This indicates that the status pursuit motivation scale for organization members has high reliability.

### Validation Study

#### Sample and Collection

In this study, exploratory factor analysis was used to preliminarily classify the motivation dimensions of membership status pursuit, but the overall fitting degree of the final factor results could not be analyzed. Therefore, re-sampling was required to verify the scale through confirmatory factors. Accordingly, a total of 450 questionnaires were distributed to knowledge-intensive enterprises in Guangdong province, Jiangsu Province, and Jiangxi Province, and 425 were recovered. We promise participants that the data obtained will only be used for academic research and will not be used for other purposes, and that the confidentiality of the questionnaire will be guaranteed. After removing questionnaires with more than three missing items or more than five consecutive identical options, 413 questionnaires were finally obtained, with an effective recovery rate of 97.2%. As can be seen from Table 4, 47.9% were male. In terms of age, 10.9% were 25 and below, 39.2% were from 26 to 35 years old, 35.4% were aged 36–45, and 14.5% were aged 46 and above. In terms of education level, 22.8% were high school or below, 26.6% had vocational high school or junior college degrees, 41.6% had undergraduate degrees, and 9% has master’s degrees or above. In terms of working years, 11.1% had worked for 1 year or less, 37% had worked 2–5 years, and 51.8% had worked more than 5 years.

**TABLE 4:** Composition of valid samples (*N* = 413).

Name	Category	Percent	Name	Category	Percentage
Gender	Male	47.9	Education	High school or below	22.8
	Female	52.1		Vocational school/College	26.6
Age	25 years old	10.9		Undergraduate	41.6
	and below				
	26–35 years old	39.2		Master degree or above	9
	35–45 years old	35.4	Work	One year or less	11.1
	Over 46 years old	14.5		Two to five years	37
				More than 5 years	51.8

#### Measures

SPSS20.0 software was used to perform KMO and Bartlett sphere tests on the data to confirm whether exploratory factor analysis could be performed. The KMO statistic is 0.8, indicating that common factors exist between variables, which is suitable for factor analysis. The Bartlett sphere test conforms to the standard of significance level of 0.05, indicating that there are common factors among correlation matrices representing the whole population, which also indicates suitability for factor analysis. Factor extraction was carried out according to the principle of feature roots being greater than 1 and the skew rotation method. After several rotations, a scale for organizational member status pursuit motivation was obtained, which consisted of two dimensions and nine items. As can be seen from Table 5, the results of exploratory factor analysis are basically consistent with the pre-set factor results. Two dimensions of prevention-focused status pursuit motivation and promotion-focused status pursuit motivation were extracted, and the total variance explanation percentage reached 62.07%. This shows that the concept of the membership status pursuit motivation scale has been preliminarily verified.

**TABLE 5:** Exploratory factor analysis results (*N* = 413).

Item	Promotion-focused	Prevention-focused
A1 I will take the initiative to share my knowledge and skills	0.698	
A3 I will overfulfill the task assigned by the leader	0.755	
A4 I will take the initiative to improve the skills required by the work	0.862	
A5 I will take the initiative to help others	0.756	
A6 I will actively participate in the activities of the company	0.686	
A9 I will be reserved in my work		0.801
A10 I will not give opinions		0.845
A11 I will take my job seriously		0.844
A12 I will make sure I have the necessary skills for my job		0.804

**FIGURE 1:**
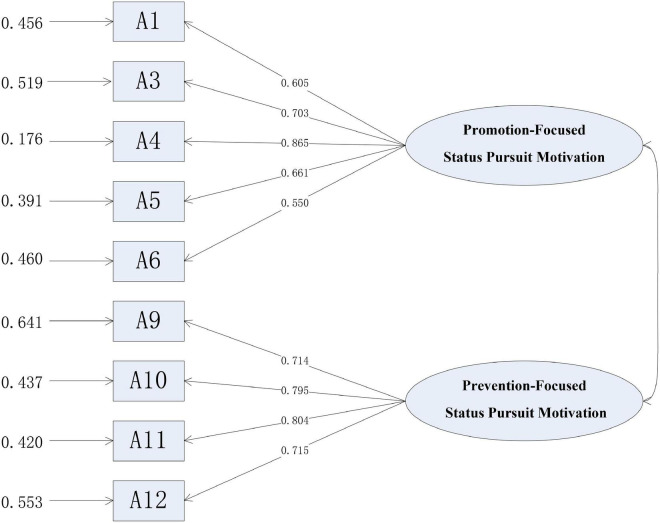
Confirmatory factor analysis.

Confirmatory research on the scale of status pursuit motivation in organization members included the violation estimation test, confirmatory factor analysis, model fit evaluation, and reliability and validity tests. Amos21.0 software was used for all standardized estimated parameter values of latent variables. It showed that the standardized fine balance of observed variables is between 0.550 and 0.865, not exceeding or too close to 1, and that the *T* value is large (see Figure 1). In addition, the index errors of all observed variables are small and without negative values, and the measurement errors are between 0.176 and 0.641, greater than 0 and not too large. These results indicate that there is no violation estimation for all observed variables.

#### Data Analysis and Result

SPSS20.0 software was used to analyze the reliability of the sample data. The results showed that the α values of each dimension of prevention-focused status pursuit motivation and promotion-focused status pursuit motivation were 0.806 and 0.842, respectively, and the α values of each dimension of the prevention-focused status pursuit motivation and the promotion-focused status pursuit motivation were decreased after deleting any item. The α value of the status pursuit motivation scale for the whole organization was 0.749, which was higher than the standard of 0.7. This indicates that the status pursuit motivation scale for organization members has high reliability.

The results of confirmatory factor analysis of the status pursuit motivation scale are shown in Figure 1. It can be seen from Figure 1 that the factor load of each item in the scale is higher than the standard value of 0.5 and reaches the level of significance, indicating that the scale has appropriate structural validity. The results of confirmatory factor analysis are shown in Table 6. It can be seen from Table 6 that the fitting effect of the two-dimensional model is good. In the status pursuit motivation scale, the combined reliability CR of promotion-focused status pursuit motivation and prevention-focused status pursuit motivation are 0.873 and 0.894, respectively; both of these are greater than 0.7. Therefore, the scale passes the combined reliability test. In addition, the AVE of promotion-focused status pursuit motivation and prevention-focused status pursuit motivation are 0.579 and 0.677, respectively; both are greater than 0.5. Therefore, the status pursuit motivation scale has good convergence validity. The latent correlation coefficient is 0.074, and its square value is 0.005. This is less than the AVE minimum value of 0.579. Lanaj et al. (2012) showed through meta-analysis that there is little correlation between promotion focus and defense focus, and our analysis results also support this conclusion. Therefore, the discriminative validity of the status pursuit motivation scale is good.

**TABLE 6:** Results of confirmatory factor analysis (*N* = 413).

Model	χ2	df	RMSEA	GFI	CFI	RMR
One-factor model	712.497	27	0.248	0.676	0.481	0.252
Two-factor model	69.923	26	0.064	0.966	0.967	0.045

## Conclusion and Discussion

For a long time, empirical research on organizational membership status has mainly been carried out within the background of Western culture. In recent years, membership status in the organization has become a topic of wide interest by scholars at home and abroad. Some scholars have explored in detail the acquisition, maintenance, and experience of organizational membership status in the workplace (Blader and Chen, 2012; Chen et al., 2012; Anicich et al., 2015; Hays and Bendersky, 2015; Smith and Magee, 2015). Membership status in organizations has gradually moved to the forefront of organizational behavior research. In China, research on membership status in organizations has gradually increased in importance. At present, scholars have examined the relationship between status and knowledge sharing and innovation, and have made a detailed review of the research on membership status in Western organizations (Liu et al., 2014, 2015; Wei and Zhang, 2014; Hu and Xie, 2015; Wang and Du, 2015; Wei et al., 2015). Hu and Xie (2015) showed that in the field of knowledge management, knowledge hiding is mainly motivated by the status preservation of organization members. Liu et al. (2013) based on different organization members’ demands for status, divided status competition motivation into dominance-based status-striving motivation and prestige-based status-striving motivation. The above studies show that status pursuit is a basic motivation of people’s activities, but status pursuit motivation is not a one-dimensional construct. Future research needs to further explore the internal structure of status pursuit motivation, so as to clarify the real relationship between status pursuit motivation and job performance and other outcome variables.

This study solves this problem to some extent. This study mainly draws the following conclusions: First, employees in organizations have different status pursuit motivations, and the previous division of status pursuit motivation into dimensions has some limitations. Second, according to regulatory focus theory, the two types of motivation can be effectively divided and explained. The status pursuit motivation scale for organization members includes two dimensions: promotion-focused status pursuit motivation and prevention-focused status pursuit motivation. The former includes five items: “I will take the initiative to share my knowledge and skills,” “I will overfulfill the tasks assigned by the leader,” “I will take the initiative to improve the skills required by the work,” “I will take the initiative to help others,” and “I will actively participate in the activities of the company.” The latter includes four items: “I will be reserved in my work,” “I will not give opinions,” “I will take my job seriously,” and “I will make sure that I have the necessary skills for the job.” Finally, we conducted three data collections on knowledge-intensive enterprises in Guangdong, Jiangsu, and Jiangxi provinces of China. Exploratory factor analysis, confirmatory factor analysis, and other statistical methods were used to analyze the data. The reliability and validity of the status pursuit motivation scale were verified. This provides a guarantee for the reliability of the scale.

The theoretical contributions of this study are as follows: First, since the existing literature fails to make a clear distinction in the dimensionality structure of the motivation for status pursuit by organization members, this study distinguishes two dimensions and defines the concept of the motivation for status pursuit by organization members on the basis of the existing results, so as to clarify the connotation of the motivation for status pursuit and enrich the existing literature on status research. Second, we developed a scale with good reliability and validity. Through a variety of statistical methods, we developed a status motivation scale that provides a clearly structured measurement tool for subsequent status-related research. Finally, the inconsistency of previous research conclusions requires us to further explore the internal structure of status pursuit motivation, so as to clarify the real relationship between status pursuit motivation and job performance, knowledge hiding, employee innovation, and other outcome variables (Gu and Peng, 2010; Cerne et al., 2014; Langfred and Moye, 2014; Huo et al., 2016; Cerne et al., 2017). According to regulatory focus theory, this study divides status pursuit motivation into prevention-focused status pursuit motivation and promotion-focused status pursuit motivation, and thus defines two tendencies of members in organizations in the pursuit of status. This helps to clarify the real relationship between status pursuit motivation and employee behavior. In addition, the research results of Lanaj et al. (2012) show through meta-analysis that there is little correlation between prevention focus and promotion focus. The conclusion of this study supports this view again.

The management implications of this study are as follows: First, managers can use the status pursuit motivation scale in the recruitment process. According to the measurement results generated by this scale, the manager can provide suitable jobs for the candidates. Then, the manager can explain to the candidate the specific work content and working environment of the position. In this way, the matching of jobs can be effectively improved. For example, the candidates with the tendency of promotion motivation can be placed in a more challenging position, while candidates with the tendency of prevention motivation can fill a detailed and serious job. Second, managers should provide employees with more flexible working styles and more job rotation opportunities. There is the possibility of mutual conversion in the motivation of status pursuit by organization members. For example, some employees who pursue promotion motivation may switch to prevention motivation as they get older. Therefore, managers should provide employees with more flexible working styles, such as giving employees the opportunity to try different positions through regular job rotation. Finally, managers should regularly evaluate the talents of the employees in the organization, and rotate and adjust the positions according to the changes in the employees’ individual motivations.

## Research Limitations and Future Research

This study also has some limitations: first, the text analysis was mainly based on the subjective coding analysis carried out by the team. In future research, qualitative analysis software can be used to analyze the data, so as to make the research results more scientific and rigorous. Second, this study failed to verify the utility of the scale, and future research can further verify the validity of the scale quantitatively. For example, knowledge, as an important political resource for gaining organizational status and power, has an inseparable relationship with status. In view of the differences in the status pursuit motivations of organization members, it is worth further verifying how the status pursuit motivations of organization members affect knowledge transfer, especially the relationship between status pursuit motivation and knowledge hiding and knowledge sharing. Finally, the three questionnaires of this study were taken from China. Whether this scale is applicable to groups outside China needs further verification.

## Data Availability Statement

The raw data supporting the conclusions of this article will be made available by the authors, without undue reservation.

## Ethics Statement

Ethical review and approval was not required for the study on human participants in accordance with the local legislation and institutional requirements. However, this study was reviewed and approved by the Jiangxi University of Finance and Economics and Hainan Normal University. All participants provided written informed consent, and they were informed of their right to withdraw from the survey at any time.

## Author Contributions

ZW: conceptualization, formal analysis, investigation, methodology, supervision, writing—original draft preparation, and funding acquisition.

## Conflict of Interest

The author declares that the research was conducted in the absence of any commercial or financial relationships that could be construed as a potential conflict of interest.

## Publisher’s Note

All claims expressed in this article are solely those of the authors and do not necessarily represent those of their affiliated organizations, or those of the publisher, the editors and the reviewers. Any product that may be evaluated in this article, or claim that may be made by its manufacturer, is not guaranteed or endorsed by the publisher.
